# A process evaluation accompanying an attempted randomized controlled trial of an evidence service for health system policymakers

**DOI:** 10.1186/s12961-015-0066-z

**Published:** 2015-12-12

**Authors:** Michael G Wilson, Jeremy M Grimshaw, R Brian Haynes, Steven E Hanna, Parminder Raina, Russell Gruen, Mathieu Ouimet, John N Lavis

**Affiliations:** McMaster Health Forum, McMaster University, Hamilton, Canada; Centre for Health Economics and Policy Analysis, McMaster University, Hamilton, Canada; Department of Clinical Epidemiology and Biostatistics, McMaster University, Hamilton, Canada; Clinical Epidemiology Program, Ottawa Hospital Research Institute, Ottawa, Canada; Department of Medicine, University of Ottawa, Ottawa, Canada; Health Information Research Unit, McMaster University, Hamilton, Canada; McMaster Evidence Review and Synthesis Centre, McMaster University, Hamilton, Canada; Lee Kong Chian School of Medicine, Nanyang Technological University, Singapore, Singapore; Department of Political Science, Université Laval, Québec, Canada; Centre de Recherche du Centre Hospitalier Universitaire de Québec, Québec, Canada; Department of Political Science, McMaster University, Hamilton, Canada; Department of Global Health and Population, Harvard School of Public Health, Cambridge, USA

## Abstract

**Background:**

We developed an evidence service that draws inputs from Health Systems Evidence (HSE), which is a comprehensive database of research evidence about governance, financial and delivery arrangements within health systems and about implementation strategies relevant to health systems. Our goal was to evaluate whether, how and why a ‘full-serve’ evidence service increases the use of synthesized research evidence by policy analysts and advisors in the Ontario Ministry of Health and Long-Term Care as compared to a ‘self-serve’ evidence service.

**Methods:**

We attempted to conduct a two-arm, 10-month randomized controlled trial (RCT), along with a follow-up qualitative process evaluation, but we terminated the RCT when we failed to reach our recruitment target. For the qualitative process evaluation we modified the original interview guide to allow us to explore the (1) factors influencing participation in the trial; (2) usage of HSE, factors explaining usage patterns, and strategies to increase usage; (3) participation in training workshops and use of other supports; and (4) views about and experiences with key HSE features.

**Results:**

We terminated the RCT given our 15% recruitment rate. Six factors were identified by those who had agreed to participate in the trial as encouraging their participation: relevance of the study to participants’ own work; familiarity with the researchers; personal view of the importance of using research evidence in policymaking; academic background; support from supervisors; and participation of colleagues. Most reported that they never, infrequently or inconsistently used HSE and suggested strategies to increase its use, including regular email reminders and employee training. However, only two participants indicated that employee training, in the form of a workshop about finding and using research evidence, had influenced their use of HSE. Most participants found HSE features to be intuitive and helpful, although registration/sign-in and some page formats (particularly the advanced search page and detailed search results page) discouraged their use or did not optimize the user experience.

**Conclusions:**

The qualitative findings informed a re-design of HSE, which allows users to more efficiently find and use research evidence about how to strengthen or reform health systems or in how to get cost-effective programs, services and drugs to those who need them. Our experience with RCT recruitment suggests the need to consider changing the unit of allocation to divisions instead of individuals within divisions, among other lessons.

**Trial registration:**

This protocol for this study is published in Implementation Science and registered with ClinicalTrials.gov (HHS/FHS REB 10–267).

**Electronic supplementary material:**

The online version of this article (doi:10.1186/s12961-015-0066-z) contains supplementary material, which is available to authorized users.

## Background

Health system policymakers make important decisions every day about the governance, financial, and delivery arrangements within which programs, services, and drugs are provided and about implementation strategies [1]. For these decisions to be evidence-informed, policymakers need timely access to the best available research evidence in a way that makes it easily retrieved (i.e. using terminology that is intuitive to them) and which allows them to rapidly scan for relevance and decision-relevant information [2,3]. For systematic reviews, which are increasingly seen as a key source of research evidence for informing the decisions made by health system policymakers [1], this means allowing for rapid scanning of how recently the search was conducted, the settings in which the included studies were conducted, and the quality of the review, as well as providing access to user-friendly summaries of the evidence whenever possible.

Policymakers also need to be alerted to the availability of new research evidence in their areas of interest to them. As outlined in the protocol for this study [4], until relatively recently no such evidence services had been developed for health system policymakers, unlike the situation for clinical and public health professionals [5,6]. To address this gap, we developed a full-serve evidence service comprised of two types of activities (efforts to facilitate ‘pull’ and ‘push’ efforts) included in a framework for supporting the use of research evidence [7]. The full-serve evidence service included (1) access to Health Systems Evidence (HSE) as a ‘one-stop shop’ for research evidence addressing questions about governance, financial and delivery arrangements within which programs, services and drugs are provided and about implementation strategies [8] (as an effort to facilitate policymakers’ efforts to ‘pull’ in research when they need it); (2) monthly email alerts about new additions to the database (a ‘push’ effort); and (3) full-text article availability (an additional effort to facilitate pull) [4]. The ‘self-serve’ evidence service consisted only of database access. Our objective was to evaluate whether (and how and why) this full-serve evidence service increased the use of synthesised research evidence by policy analysts and advisors in the Ontario Ministry of Health and Long-Term Care as compared to a ‘self-serve’ evidence service.

## Methods

As detailed in our published study protocol [4], we planned for a two-arm randomized controlled trial (RCT), along with a follow-up qualitative process evaluation to explore how and why the ‘full-serve’ evidence service did or did not work. We invited all policy analysts and advisors from a purposively selected division in the Ontario Ministry of Health and Long-Term Care, which deals with health systems issues on a regular basis. For those agreeing to participate, we planned to use a stratified randomized design to assign participants to receive either the ‘full-serve’ or ‘self-serve’ evidence service. The trial was to be conducted over a 10-month period consisting of three phases: (1) a 2-month baseline period where all participants would receive the ‘self-serve’ evidence service; (2) a 6-month intervention period where the intervention group would receive the ‘full-serve’ evidence service; and (3) a 2-month cross-over period where all participants would receive the ‘full-serve’ evidence. We planned to measure the mean number of site visits/month/user between baseline and the end of the intervention period as the primary outcome and participants’ intention to use research evidence as the secondary outcome. For the qualitative process evaluation, our original plan was to conduct one-on-one semi-structured interviews with participants (15 from the intervention group and 15 from the control group) on their views about and experiences with the evidence service they received, how and why it was helpful (or not) in their work, what features were most and least helpful and why, and recommendations for next steps.

However, as we report in the results section in more detail, we terminated the RCT when we failed to reach our recruitment target of 148, which was the number of policy analysts and advisors working in the purposively sampled division at the time of publishing the protocol (this changed to 138 by the time we started recruitment). After the termination of the trial, we revised the original interview guide (provided in Additional file 1) to explore actual and potential participants’ reasons for deciding to participate or not in the trial and their views about whether, how and why HSE has been helpful in their work, what features are most and least helpful and why, and recommendations for how to improve it. Ethics approvals for the study and its amended version were received from the HHS/FHS REB at McMaster University (protocol #10-267). Written consent was obtained from each study participant prior to participating in the interview.

### Study population and recruitment

We derived a sample frame for the process evaluation by reviewing the sample of 138 policy analysts and advisors who were originally invited to participate in the RCT and identified those still in the same division (Health System Strategy and Policy Division) from which the sample was drawn. Using the list of 78 individuals who met these criteria, we selected a purposive sample of 30 policy analysts and advisors, of whom 15 had originally agreed to participate in the trial and 15 had not. In selecting each half of the sample, we included a mix of participants from different branches and units within the division and from different types of positions (e.g. senior and junior policy analysts and advisors). As reported in the results, all of those who participated in the qualitative interviews were those who had agreed to participate in the RCT. Based on this, we determined that additional sampling would be highly unlikely to yield additional participants given that, of those remaining in the sample frame, most had not responded and some had declined the invitation for the RCT phase of the study.

### Data collection

We invited participants by email to take part in semi-structured telephone interviews. The interviews were conducted by MGW using an interview guide and audio-taped the interviews, and we transcribed the interviews verbatim. The first part of the interview guide explored the factors influencing participation in the trial. We included an initial set of probes for these factors, such as the time commitment involved, concerns about their HSE usage being monitored, and the trial’s perceived relevance to their work. The second part of the interview guide explored (1) participants’ usage of HSE, factors explaining usage patterns, and strategies to increase usage; (2) participation in training workshops and use of other supports; and (3) views about and experiences with key HSE features. We asked explicitly about eight of HSE’s key features: (1) registration and sign-in; (2) open search; (3) advanced search; (4) search overview page; (5) detailed search results page; (6) links to one-page summaries; (7) monthly evidence service; and (8) supplementary material/portals. We added the last probe to solicit feedback about the Evidence-Informed Healthcare Renewal Portal that was added after the trial was terminated and provides a set of policy-relevant documents related to healthcare renewal in Canada [9]. Prior to the interviews, we asked participants to use HSE and familiarize themselves with its features. In addition, during the interviews, the participant and the interviewer typically signed into the database at the same time so that ‘real-time’ feedback could be received.

### Data management and analysis

We used a constant comparative approach [10] to data analysis, whereby we identified key themes and findings emerging from successive interviews and refined the interview guide as needed following each interview to address these topics in subsequent interviews (which included adjusting our initial list of probes where needed). This strategy allowed us to gather additional feedback on these themes from later interviewees. Upon completing the interviews, we summarized the findings along with illustrative quotes in four key domains: (1) factors influencing participation in the trial; (2) usage of HSE, factors explaining usage patterns, and strategies to increase usage; (3) participation in training workshops and use of other supports; and (4) views about and experiences with key HSE features. We did not use qualitative analysis software such as NVivo to analyse the results given the small number of interviews conducted and because the interviews were brief. Instead, we used the verbatim transcripts to develop structured summaries for each interview according to our areas of interest outlined above.

## Results

### Randomized controlled trial (RCT)

From the 138 policy analysts and advisors who were working in the division at the time and hence invited to participate in the trial, only 59 responded to either of our three waves of study invitations (a 43% response rate). Of these, 21 agreed to participate (a 15% trial recruitment rate) and 38 declined. Of the 38 who declined to participate, 16 were still in the same ministry but no longer in the same division, nine did not provide a reason, seven were on short-term contract, retiring or going on leave, and six had moved to a different ministry. Given our failure to reach our recruitment target in the division addressing health system issues most directly, we terminated the trial.

### Qualitative process evaluation

Of the 30 policy analysts we invited to participate in the qualitative process evaluation, nine agreed to participate, of whom seven had agreed to participate in the trial and two had not. While limited in size, the sample included participants from six different units and covered a range of sectors (including acute care, community care, and health protection and promotion), populations (e.g. people living with mental health and addictions) and decision support (e.g. economic analysis and evidence synthesis). However, the sample was not as balanced in terms of level of position with eight participants in senior analyst roles and one in a junior role.

#### Factors influencing participation in the trial

While two of the study participants had not agreed to participate in the trial according to our study records, during the interview all nine reported having agreed to participate in the trial. Participants cited a number of factors that encouraged their participation in the trial, which included the relevance of the trial to their own work; familiarity with the researchers; personal view of the importance of using research evidence in policymaking; academic background; support from supervisors and the Assistant Deputy Minister; and the participation of colleagues.

Three participants also reported that the time commitment had been a concern for them, and many noted that there are often a large number of tasks to accomplish and non-prioritized tasks are often ignored. For example, one participant noted that they “…*think people have a variety of different pressures on their time here and they don’t understand the importance of research so they put it down lower on the priority list*.” The same participant indicated that, given these pressures, “*to have the manager say this was actually something that is really a priority, helped us to prioritize that as something to do*.”

One participant expressed concern about having to be careful what policy analysts and advisors said as a representative of the ministry, but none of the study participants were concerned about their HSE usage being monitored as part of the trial. In general, they suggested that trials such as this one are “*a good thing for the policy and planning community*” as they help to ensure that policymakers “*get better access to evidence and tools*.”

#### Usage of HSE, factors explaining usage patterns, and strategies to increase usage

Six of the participants had used HSE before the interview, but these same participants indicated that they used it infrequently or inconsistently. Despite this, almost all of the participants (n = 7) stated that they felt it would be useful for their work-related projects. Two participants indicated that HSE was a helpful resource because it helps policymakers meet their need for finding relevant research evidence to support and inform the development of policy initiatives. For example, one participant noted that “*with any policy initiative you have, especially with the Ministry of Health, there’s a huge push to have evidence. So when you’re writing different types of documents to support initiatives, you need to have options and they need to be supported with evidence*.”

When asked for reasons why HSE was used infrequently or inconsistently, all participants indicated that they already use multiple databases and one participant specifically indicated that they often prefer to stick to the ones they know. In addition, three participants noted that they are typically not required to directly seek research evidence themselves because units in the ministry already do this for them. One participant said that rather than having different databases addressing different healthcare topics (e.g. only about health systems), it would be better to more generally include evidence across healthcare boundaries. Specifically, this participant indicated that:

“*It frustrates me to no end that we continue to have all of these different partitioning*[s] *of evidence. Which is both good, because it allows us to focus but also bad because there is* […] *much less cross-boundary searching that we can do. I always have to end up going back to the general databases to search* […] *being able to specifically say, for example, I want to look for something that crosses the boundaries of public health and primary care and community care, that would be fantastic.*”

When asked about what could be done to increase their use of HSE, four common themes emerged, namely reminders about the existence of the site, such as the monthly evidence service; encouragement of its use from management in general and supervisors in particular; incorporation into employee training; and promotion on the Ministry’s website.

One participant highlighted that:

“*if there’s buy in from senior management here to help promote a tool like this or to encourage people to use it, that’s also a helpful way in terms of getting the attention of people who are doing policy work because often, it takes people getting direction from someone above to motivate them sometimes”*

#### Participation in training workshops and use of other supports

Six of the participants had participated in workshops on finding and using research evidence provided to ministry staff in which HSE is profiled as a key source for research evidence to address questions related to health systems. Two others had never participated but had read the material, and one had not participated or read the material. Of those who had participated, two indicated that they thought it had an impact on their use of HSE. Another two participants said the workshop was helpful because it taught them about quality ratings (like the ones provided in HSE) and another participant noted that the workshop helped them learn about the different search tools. Others indicated that attending the workshop did not influence their use of the website. For example, one said: “*I actually went to* [the] *workshop and he* [the facilitator] *probably talked about it and then just went, you know, out the other ear*.” None of the participants had watched the tutorial videos provided through HSE, but three stated they thought they could be helpful. Two participants also said the tips on the site itself were useful.

#### Views about and experiences with key HSE features

In general, most participants (six out of nine) said they found the features of HSE to be intuitive. Some said they found the information it contains more relevant for policymakers than most other search engines. One participant indicated that the documents found in HSE “*were all consistently very high quality compared to if I did my usual bibliography searches on the literature databases*.”

Table 1 summarises the feedback received regarding each of eight key features of HSE along with illustrative quotes. In general, participants indicated that searching is intuitive; having multiple options to conduct searches (e.g. by searching one or more topic categories) allows for greater flexibility and functionality; providing an overview of the number and types documents retrieved by a search and the ability to limit the results to specific types of documents on the search results overview page is helpful; providing a monthly evidence service is important for providing alerts about new research evidence; including access to a diversity of document types is helpful; and having access to a database focused on policy documents related to the Canadian healthcare system (the Evidence-Informed Healthcare Renewal portal) is an important source of additional evidence. However, several participants also indicated that the requirement to register and then sign-in before searching HSE either discouraged their use of the database and/or could discourage its use by others. Others found the layout of the advanced search and the results pages to be confusing and emphasized the need to more effectively highlight the most important information (e.g. the main search functionality on the advanced search page and the titles in the results page).

**Table 1:** **Views about and experiences with key Health Systems Evidence (HSE) features**

**HSE feature**	**Key findings**	**Illustrative quotes**
Registration and sign-in	• Four participants reported feeling that the registration and sign-in decreased their willingness to use the database, while others said that they did not consider it a nuisance, especially since many sites now require sign-in • Five participants reported that having to remember a password could discourage use, but some noted that this challenge could be overcome by the option to save passwords on a computer and by frequent use of the site • Three participants expressed confusion over the purpose and value of registration and sign-in and two stated they would prefer a one-time only registration for mailing purposes, but without the repeated need to sign-in	“I don’t know why there’s a need to even sign in. That’s what I’m saying. I don’t know why, unless you’re trying to track the people who are using it somehow” “It does not limit my willingness to use it but I find it a bit annoying because I thought when I signed up the first time, it says a one-time registration so I assumed that I would sign in one time and then from then on, it would recognize my computer”
Open (basic) search page	• Four participants stated that they found the open search page intuitive • Participants generally felt it was simple to use and user friendly, and liked being presented with it before deciding to move to the advanced search page • In discussing the open search page, participants also highlighted their preference for searching with keywords instead of having to do a very specific search immediately	“For the most part I think keywords are just the way we search. It’s just part of the way you do it normal life, so I find normal searching, keyword searching, to be the easiest for me and the most intuitive”
Advanced search page	• Participants noted that they liked the ability to search by health system topic and combine those searches with an open search (i.e. using keywords) and limits • Three participants thought it was organized intuitively, indicating that the page did not look ‘too busy’ given that different sections of the page can be expanded to display more functionality • Many expressed concerns over the layout, with seven stating they found some of the functions and categories difficult to find and three saying they were unsure where to enter search terms • The most frequently requested changes were to add an option to limit a search to specific countries and to high-income countries (and not just low- and middle-income countries)	“if you’re starting from that main page where there’s just the one search field and then you click on advanced searching, I feel like it should keep that main search field there at the top of the page and then when you click on expand, it should expand below that and have the four options, the tick boxes, below that” “if I had the option of saying you know tick, tick, tick, I want these countries, that would be amazing” and “because [the ministry] does a lot of comparative work with the Commonwealth countries”
Search results overview page	• Eight participants said that they liked the search results overview page, which appears after completing a search but before the detailed search results page is provided • Participants like the opportunity to select the types of documents they would like to have appear in the detailed search results • One participant noted they would prefer if this page could be selected as an optional intermediate step and two participants thought the organization was confusing • Six of the participants had never noticed the option to re-run the search in EvidenceUpdates or in PubMed (using the search hedges for health services research), or at least felt it was too hard to find and needed to be made more prominent • Upon learning that these features were available, participants said that having the option to re-run their search in EvidenceUpdates and PubMed was helpful	“…really loved the way that it categorized the results in all those little squares so I knew immediately what type of evidence I was going to get” “Nobody’s going to see that stuff [option to rerun searches in EvidenceUpdates and PubMed]. You’ve got a lot of little notes. What people do is they ignore the little notes at the bottom”
Detailed search results page	• Four participants felt that the results page looked ‘too busy’ and had too much information • Many participants highlighted information that they felt was most important and therefore needed to be made more prominent by moving it to the left columns (to facilitate screening of information across rows) and ensuring the most important information has contrasting font (to make it more noticeable) • Most participants suggested that the title should be made most prominent and to have it link directly to either the one-page summary or to the full-text • Feedback about the priority that should be accorded to the other columns was mixed, with each of the remaining columns (countries in which studies were conducted or that are the focus of the document, the last year the literature was searched or year published, quality rating, type of question, and type of document) being suggested by at least one participant as important to be emphasized more in the results table	“The page is too much information. You’re just on information overload on the page. You need three or four columns, something like that” “When I click on the title, I expect that it will take me to your one-page summary. But I think that if you had ‘full text report’ itself, if you could just have the title linked to that directly so that rather than having to click on Show Links and then Click on Full Text Report, title itself just hyperlinked directly to the Full Text Report, that would just eliminate an extra step”
Links to one-page summaries	• Three participants indicated that they found the one-page summaries to be helpful with the main reasons being that it offered a pre-digested form of information from the paper and could easily be saved or printed to be viewed later • Three other participants felt that the material in the summaries was too similar to the results page and some also felt that, due to time pressures they are under, they would not usually take the time to read a summary as they were more concerned with finding the full-text version of the document	“just having adjusted information; I just find it to be great. It’s pre-digested information which is usually helpful in cases where I’m trying to summarize” “as a policy person with one hour time limit, to get something to senior management, I wouldn’t look at it” “have the starting at the last year literature search, quality rating, all of that stuff down to the citation up closer to the top and then put the type of document, type of question, health system topics, domain, etc. lower down”
Monthly evidence service	• Only two participants reported receiving the monthly evidence service, however, five stated that they thought they would be valuable as it is convenient to not have to go look for research and to remind them to use HSE	“if people knew about this type of website where you could have information sent directly to you without having to keep going in and do searches, I think probably would be very helpful. That’s one of the main ways that I get information”
Types of documents	• Participants said they liked the diversity of documents in HSE, particularly the economic evaluations and documents related to health care renewal. • Two participants also said they liked that ongoing systematic reviews (i.e. systematic review protocols) and systematic reviews being planned (i.e. registered systematic review titles) were indexed in HSE	“Knowing that someone is going to be doing a systematic review in cases where we can’t find information […] because we consider our finding to be there hasn’t been much research, so it’s nice to be able to say that someone is looking at this”
Supplementary material/portals	• Six participants stated that they found the Evidence-Informed Healthcare Renewal (EIHR) Portal helpful and relevant to their work because it makes it easy to examine what other jurisdictions are doing, as well as being helpful to give them access to grey literature given that academic/peer-reviewed literature often does not address topics that meet their needs and interests • Despite finding it useful, three participants stated they had never noticed the EIHR Portal before	“because there are topics where the academic literature isn’t as pertinent and documents from the grey literature are more useful but it’s harder to find those documents” “…might be an easier term to call it rather than evidence informed healthcare renewal. It just sounds really jargony and doesn’t really apply to what you just told me, in my opinion”

## Discussion

### Principal findings

We terminated the RCT – to our knowledge, the first attempt to evaluate the effects of an evidence service specifically designed to support health system policymakers in finding and using research evidence using such a design – given our 15% trial recruitment rate. Six factors were identified by those who had agreed to participate in the trial as encouraging their participation: relevance of the study to participants’ own work; familiarity with the researchers; personal view of the importance of using research evidence in policymaking; academic background; support from supervisors; and participation of colleagues. Most participants reported that they never, infrequently or inconsistently used HSE and suggested several strategies to increase its use, including regular email reminders and employee training. However, only two participants indicated that employee training, in the form of a workshop about finding and using research evidence, had influenced their use of HSE. Most participants found HSE features to be intuitive and helpful, although registration/sign-in and some page formats (particularly the advanced search page and detailed search results page) discouraged their use of HSE or did not optimize the user experience.

### Strengths and limitations

The principal strength of our study is that we received in-depth feedback from a sample of policy analysts and advisors (a key target audience for us) about trial participation and HSE features and we have acted on many of their suggestions for improving HSE. The principal limitations of our study relate to sample size, both in terms of the very low recruitment rate for the trial (which resulted in its termination) and in terms of the somewhat low recruitment rate for the qualitative process evaluation, particularly among those who did not volunteer for the trial. In addition, it is likely that many of those who agreed to participate in the qualitative interviews were those already keen to use HSE in their work, which may have limited our ability to identify divergent views. However, we did observe the recurrence of common themes in each interview and with participants from a diverse set of units in the division, which leads us to believe that, if data saturation was not reached in all domains (e.g. because of potentially not reaching a sufficiently balanced sample that provided divergent views), we were likely close to achieving it.

### Implications

We have identified implications related to future trial recruitment and for efforts to support the use of research evidence by policymakers. For future trial recruitment, our study provides a helpful reflection about how best to recruit policy analysts and advisors in a trial within a particular division of a health ministry even when a team has the explicit support of an Assistant Deputy Minister, who is the divisional head, in the form of both a formal letter attached to our invitation and an internal email that was sent to staff of the division. One particular point for reflection is whether our ‘unit of allocation’ for the intervention should have been divisions and not individuals within a division. Policy development is a complex process that depends on the efforts of many policy analysts and its use at the individual level is therefore likely to vary depending on policy priorities at a given point in time. As a result, the more meaningful unit of analysis could be the use of an evidence service in a division, which would more accurately capture this variability at the individual level. In other words, the focus would instead be on studying the effect of our evidence service on the use of research evidence by all policy analysts and advisors within their division as the more meaningful level of outcomes.

In addition to modifying our approach to analysis, changing the unit of allocation has significant implications for the levels at which informed consent should be obtained for a cluster RCT [11]. Specifically, changing the unit of allocation in this way would mean seeking consent at the level of the division (i.e. the Assistant Deputy Minister), plus having either a waiver of consent or additional informed consent at the level of individual participants. For example, one possible approach could be to include two divisions per Canadian province that are then randomly assigned to receive the ‘full-serve’ or ‘self-serve’ evidence service. A limitation would be a lack of statistical power, but such a design would add to a larger body of knowledge that could be replicated and then included in meta-analyses.

For efforts to support the use of research evidence, our study supports others’ findings that essential components of such efforts include the need to ensure timely, multi-faceted yet focused communication [2,12-14] and to enhance interaction between the producers and users of research evidence [2,12,13]. For example, a recent Cochrane review of interventions to improve the use of systematic reviews in decision-making by health system managers, policymakers and clinicians, indicated that simple and clear messages are best in communicating results from systematic reviews [14]. In addition, the same review indicated that multi-faceted approaches (e.g. ‘push’ efforts combined with efforts to facilitate ‘pull’ such as capacity building) are needed when the goal is not only to communicate results to inform policy and practice but also to more generally increase awareness and knowledge about the importance of using systematic reviews and to build skills for using them to inform policy and practice [14].

### Study impact

Based on the feedback received about HSE, we made several modifications to enhance the functionality of the advanced search page and the detailed search results page. On the advanced search page, we modified the layout to better highlight the available search functionality. Specifically, we have incorporated a short descriptive header for the three primary groupings of search functionalities (‘topics’, ‘open search’ and ‘limits’), which are followed by a one-line description of what each can be used to do. In addition, we now highlight that the Evidence-Informed Healthcare Renewal Portal contains policy documents related to healthcare renewal in Canada. Next, we incorporated two new search limits. The first limit allows users to limit their search to documents with either a general focus (i.e. an intervention that is broadly applied across settings, populations and diseases, such as case management) or a specific focus (i.e. an intervention that is primarily focused on a single setting, a specific population or a specific disease such as case management for teenagers with diabetes in the United Kingdom). The second limit allows users to search for documents that either focus on a particular country (or countries) or include at least one study that was conducted in a country (or countries).

On the detailed search results page, we removed the columns outlining the type of question and the health system topics addressed, which were both identified by most participants as not being helpful when scanning results for relevant documents. We also now list the document title in the first column (instead of the type of document), which is then hyperlinked to the one-page summary, uses bolded text in a different colour (to make it stand out on the page) and notes whether the document has a general or specific focus in an icon below the title.

## Conclusion

The qualitative data we collected has informed the redesign of HSE, which will allow users to more efficiently find and use research evidence about how to strengthen or reform health systems or in how to get cost-effective programs, services and drugs to those who need them. We plan to continue to conduct periodic user testing to ensure that HSE is an optimal tool to identify research evidence about health systems, as well as to explore ways to increase trial recruitment rates among policy analysts, and more generally approaches to future evaluations.

## Additional file

Additional file 1:
**Interview guide for qualitative process evaluation.** (DOCX 17 kb)

## Abbreviations

HSE: Health Systems Evidence
RCT: Randomized clinical trial

## References

[1] Lavis JN (2009). How can we support the use of systematic reviews in policymaking?. PLoS Med.

[2] Lavis JN, Davies HTO, Oxman AD, Denis J-L, Golden-Biddle K, Ferlie E (2005). Towards systematic reviews that inform health care management and policy-making. J Health Serv Res Pol.

[3] Lavis JN, Davies HTO, Gruen RL (2006). Working within and beyond the Cochrane Collaboration to make systematic reviews more useful to healthcare managers and policy makers. Healthcare Policy.

[4] Lavis JN, Wilson MG, Grimshaw J, Haynes RB, Hanna S, Raina P (2011). Effects of an evidence service on healthcare policymakers’ use research evidence: a protocol for a randomized controlled trial. Implement Sci.

[5] Haynes RB, Cotoi C, Holland J, Walters L, Wilczynski N, Jedraszewski D (2006). Second-order peer review of the medical literature for clinical practitioners. JAMA.

[6] Dobbins M, DeCorby K, Robeson P, Husson H, Tirilis D, Greco L (2010). A knowledge management tool for public health: health-evidence.ca. BMC Public Health.

[7] Lavis JN, Lomas J, Hamid M, Sewankambo NK (2006). Assessing country-level efforts to link research to action. Bull World Health Organ.

[8] Wilson MG, Moat KA, Lavis JN (2013). The global stock of research evidence relevant to health systems policymaking. Health Res Pol Syst.

[9] Kowalewski K, Lavis JN, Wilson MG, Carter N (2014). Supporting evidence-informed health policymaking: the development and contents of an online repository of policy-relevant documents addressing healthcare renewal in Canada. Healthcare Policy.

[10] Boeije H (2002). A purposeful approach to the constant comparative methods in the analysis of qualitative interviews. Quality Quantity.

[11] Weijer C, Grimshaw JM, Eccles MP, McRae AD, White A, Brehaut JC (2012). The Ottawa statement on the ethical design and conduct of cluster randomized trials. PLoS Med.

[12] Oliver K, Innvar S, Lorenc T, Woodman J, Thomas J (2014). A systematic review of barriers to and facilitators of the use of evidence by policymakers. BMC Health Serv Res.

[13] Lavis JN, Catallo C (2013). Bridging the worlds of research and policy in European health systems.

[14] Murthy L, Shepperd S, Clarke MJ, Garner SE, Lavis JN, Perrier L (2012). Interventions to improve the use of systematic reviews in decision-making by health system managers, policy makers and clinicians. Cochrane Database Syst Rev.

