# Supplementary material for: A process evaluation accompanying an attempted randomized controlled trial of an evidence service for health system policymakers
Source: Health Res Policy Syst. 2015 Dec 12;13:78. doi: 10.1186/s12961-015-0066-z (PMC4677046; doi:10.1186/s12961-015-0066-z)
Supplement: Additional file 1: — Interview guide for qualitative process evaluation. (DOCX 17 kb) [file 12961_2015_66_MOESM1_ESM.docx]

**Additional File 1: Interview guide for qualitative process evaluation**

Effects of an evidence service on health system policymakers’ use of research evidence:

A randomized controlled trial

**Interview guide**

17 April 2012

Thank you for agreeing to participate in this interview. We appreciate you taking the time out of your busy schedule to provide us with feedback about Health Systems Evidence and about the recruitment phase of a randomized controlled trial of its effects. Before proceeding, I would like to confirm that I did receive your signed consent form. If at any time you wish to stop the interview, please let me know and we can stop as well as delete the audio recording and any notes that I have taken.

In this study, we are striving to understand our experiences with a trial of effects and policymakers’ views about and experiences with Health Systems Evidence. In particular, during the interview I would like to discuss our findings from the recruitment phase of the trial as well as your views about and experiences with Health Systems Evidence, including whether and how helpful it has been in your work, why it has been helpful (or not helpful), what aspects you have found to be most and least helpful and why, and recommendations for how to improve it.

**Questions**

Do you have any questions before proceeding with the interview?

As you may recall, in early 2010 we invited you to participate in a randomized controlled trial evaluating the effects of an evidence service on research use by policy analysts’ and advisors’ use of research evidence. As part of the recruitment, we sent three invitations, which contained a letter of support from the Assistant Deputy Minister of the Health System Policy and Strategy Division. In total we invited 138 policy analysts and advisors of which only 59 responded, which is a 43% response rate. From those that responded, only 21 agreed to participate and we had to terminate the trial due to insufficient sample size.

As a follow-up to the trial, we would like to get feedback about the factors that led you to participate/not participate in the trial so that we can improve upon future efforts to conduct similar evaluations of knowledge translation interventions.

- Could you comment on why decided to participate/not participate?
- [prompts for factors that may have impacted their participation]
  - Time commitment/too many competing demands?
  - Clarity of the purpose of the trial
  - Concerns about personal usage of Health Systems Evidence being monitored
  - Relevance of the intervention – the evidence service – to their work

Could you describe your general impressions of Health Systems Evidence? Is the design and content of Health Systems Evidence intuitive? Why or why not?

Had you used Health Systems Evidence before we sent you link to it before the interview?

- If yes -
  - Have you used it to find and use synthesized research evidence for any specific projects that you have worked on within the last year?
    - If so, could you provide an example of how you used Health Systems Evidence and how it was helpful?
    - If not, do you think you will use it for future project? Why or why not?
  - Have you found it to be helpful in your work? Why or why not?
- If no –
  - Were you aware of it as a resource prior to this interview?
  - Do you think it could be a helpful resource for you to find and use research evidence in the future? Why or why not?
  - In what ways do you think it could be helpful?

Have you previously participated in the workshop on finding and using research evidence that John Lavis and his colleagues periodically deliver at the Ontario Ministry of Health and Long-Term Care? Do you think it had any impact on whether and how you have used Health Systems Evidence?

What aspects of Health Systems Evidence do you find to be the most helpful? Least helpful?

- Does the one-time registration and need to sign-in each time you want to use Health Systems Evidence limit your willingness to use it as a resource? Why or why not?
- Have you accessed any of the resources listed in the ‘Background’, ‘Search tips’ or ‘Tools’ boxes on the Open search page?
  - If yes –
    - Did you find them helpful? Why or why not?
    - Did you view the video tutorial? (If yes, was it helpful?)
  - If not, why not?
- Do you find the open search intuitive to use? Why or why not?
- Do you find the advanced search to be intuitive? Why or why not?
  - How would you approach conducting a search on this page?
  - What features did you find to be the most and least helpful and why?
  - Is being able to search by health system topic a helpful feature? Why or why not?
  - Do you think the space to enter search terms is intuitive to use?
  - Do you find the limits to be a helpful search feature? Why or why not? Are there any other limits that you would like to see included?
  - Do you find the documents related to healthcare renewal in Canada to be a helpful additional source of evidence? Why or why not?
- Do you find the search overview intuitive? Why or why not?
  - Do you like the ability to view the results for each type of document and then select the types of documents you would like to view?
  - Is the feature to re-run your search in Evidence Updates or in PubMed using its search filters for Health Services Research a helpful feature? Why or why not? Have you used this feature? If so, did the searches seem to identify research evidence that was relevant to your search?

Do you find the search results page to be intuitive and helpful to find and use synthesized research evidence? For example, how helpful was information about:

- type of review
- type of question addressed
- health system arrangement or implementation strategy addressed
- title of the review
- last year searched/year published
- quality rating
- countries in which included studies were conducted
- links to user-friendly summaries, scientific abstracts, full-text review (when publicly available) and related documents in Health Systems Evidence

Do you find the one-page summaries to be helpful? In what way are they helpful (or not)?

Do you receive the monthly evidence service provided by Health Systems Evidence?

- If yes –
  - Do you find it to be a helpful feature? Why or why not?
  - Is there something specific about the evidence service that was helpful?
- If no –
  - Do you think it is something that would be helpful for you in the future? Why or why not?
- What is your impression of the upper limit placed on the number of categories you can select as your areas of interest for the evidence service? Do you find this to be limiting?

We have recently added new types of documents to Health Systems Evidence, which include economic evaluations, health reform descriptions, health system descriptions and documents related to healthcare renewal in Canada

- Do you think these are helpful sources of research evidence and information for supporting evidence-informed policy?
  - Why or why not?
  - In what ways do you think they would be helpful?
  - Is there a specific type of document that would be particularly helpful for you? Why?

In general, do you think Health Systems Evidence is a helpful tool for policymakers to find and use research evidence to support evidence-informed policy? Why or why not?

Do you have any further ideas or recommendations for how we could improve upon Health Systems Evidence?
